# Switching from intravenous to subcutaneous infliximab in patients with immune mediated diseases in clinical remission

**DOI:** 10.3389/fmed.2025.1583401

**Published:** 2025-05-16

**Authors:** Nikos Viazis, Anastasios Karamanakos, Konstantinos Mousourakis, Angeliki Christidou, Fotios Fousekis, Konstantinos Mpakogiannis, Anastasios Koukoudis, Konstantinos Katsanos, Dimitrios Christodoulou, Myrto Cheila, Maria Tzouvala, Eirini Zacharopoulou, Maria Palatianou, Olga Giouleme, Anastasia Katsoula, Christos Liatsos, Nikolaos Kyriakos, Evi Zampeli, Evgenia Papathanasiou, Angeliki Theodoropoulou, Konstantinos Karmiris, Ioannis Psaroudakis, George Tribonias, Souzanna Gazi, Evangelia Mole, Theodoros Dimitroulas, Christos Koutsianas, Dimitris Vassilopoulos, George E. Fragoulis, Nikos Michalakeas, Charalampos Papagoras, Pantelis Panagakis, Marina Papoutsaki, Vasiliki Chasapi, Alexandros Stratigos, George Katsikas

**Affiliations:** ^1^Gastroenterology Department, Evangelismos General Hospital, Athens, Greece; ^2^Rheumatology Department, Evangelismos General Hospital, Athens, Greece; ^3^Gastroenterology Department, University Hospital of Ioannina, Ioannina, Greece; ^4^Gastroenterology Department, General Hospital Nikaia Piraeus, “Agios Panteleimon”, Athens, Greece; ^5^Gastroenterology Department, Second Propedeutic Department of Internal Medicine, Hippokration General Hospital, Thessaloniki, Greece; ^6^Gastroenterology Department, 401 General Military Hospital of Athens, Thessaloniki, Greece; ^7^Gastroenterology Department, General Hospital Alexandra, Heraklion, Greece; ^8^Gastroenterology Department, Venizeleio General Hospital, Heraklion, Greece; ^9^Gastroenterology Department, Erythros Stavros, Athens, Greece; ^10^Rheumatology Department, KAT Athens, Greece; ^11^Rheumatology Department, General Hospital Ippokrateio Thessaloniki, Thessaloniki, Greece; ^12^Rheumatology Department Ippokrateio Athens, Athens, Greece; ^13^Rheumatology Department, Joint Academic Rheumatology Centre, “Laiko” General Hospital, Athens, Greece; ^14^First Department of Internal Medicine, University Hospital of Alexandroupolis, Democritus University of Thrace, Alexandroupolis, Greece; ^15^Dermatology Department, Andreas Syggros Hospital, Athens, Greece; ^16^Department of Dermatology, Andreas Syggros Hospital, Athens, Greece

**Keywords:** ulcerrative colitis, Crohn disease, infliximab (ifx), spondyloarthritis, psoriatic arthritis, psoriasis, switch

## Abstract

**Aim:**

To report on the efficacy and safety of elective switching from intravenously (IV) to subcutaneously (SC) administered Infliximab (IFX) in patients with immune mediated diseases.

**Methods:**

Retrospective analysis of patients with Crohn’s disease (CD), Ulcerative Colitis (UC), Spondyloarthritis (SpA), Rheumatoid Arthritis (RA), Psoriatic Arthritis (PsA) and chronic plaque Psoriasis (PsO) who were receiving IFX-IV for maintenance of remission in tertiary referral centers and were switched to IFX-SC based on their physician’s choice. All patients with gastrointestinal and skin diseases were in clinical remission, while those with musculoskeletal disease had inactive disease or low disease activity. The primary endpoint was disease deterioration during a follow up period, of at least 6 months, according to disease specific composite measures.

**Results:**

Between April 2023 and April 2024, a total of 344 patients (CD = 136, UC = 62, SpA = 52, PsA = 38, RA = 7, PsO = 44, co-existence of more than one disease = 5) were switched from IFX-IV to IFX-SC. After a mean±SD follow up period of 8 ± 4 months, 12 patients (3.5%) discontinued treatment with IFX-SC. Five of them (1.5%) because of disease worsening and the remaining 7 (2.0%) because of the occurrence of side effects. All 332 other patients (96.5%) showed favorable response, none of them requested an unscheduled visit, or developed an adverse event (clinical or laboratory) or needed escalation of treatment.

**Conclusion:**

Elective switching from IFX-IV to IFX-SC seems to be an effective and safe approach in real-world every day clinical practice to maintain long-term clinical remission, inactive disease or low disease activity in patients with immune-mediated diseases.

## Introduction

Infliximab (IFX) is a chimeric anti-TNFa IgG1 antibody approved for the treatment of immune mediated diseases, namely Crohn’s disease (CD), ulcerative colitis (UC), spondyloarthritis (SpA), rheumatoid arthritis (RA), psoriatic arthritis (PsA) and chronic plaque psoriasis (PsO). IFX has revolutionized the management of these diseases and has substantially improved patients’ management (1). Given the fact that the patent for the IFX originator (Remicade®; MSD, Greece) expired in 2015 and its biosimilars CT-P13 (Remsima®; Inflectra®) and PF-06438179/GP1111 (Zessly®) were approved by the European Medicines Agency in 2016 and 2018 respectively, several patients received IFX biosimilars in Greece for the management of their immune mediated diseases since then (2, 3). Furthermore, since 2021, the availability of CT-P13 in a subcutaneous formulation (Remsima®), that can be self-administered at home (after adequate training), has the potential to both improve patient convenience and reduce the burden on the healthcare system (4).

The initial approval of CT-P13 SC, for use in the treatment of RA in adults, was based on the findings of a randomized, double-blind phase I/III trial which demonstrated the non-inferiority of CT-P13 SC administered every 2 weeks to intravenous CT-P13 administered every 8 weeks in reducing disease activity in patients with active RA (5). Subsequently, based on pharmacokinetic data in patients with inflammatory bowel disease (IBD), CT-P13 SC has also been approved in the EU for use in the treatment of CD, UC and, by extrapolation, SpA, PsA and PsO, in adults (6).

In patients initiating IFX, the switch to SC formulation can be performed starting from week 6, after two infusions at week 0 and 2. In those who are already in the maintenance phase with IFX-IV, the switch can be performed at any time, based on the treating physician’s choice. However, there is no commonly accepted strategy regarding the switch from IFX IV to SC in clinical practice and real-world data on safety and efficacy of switching from one formulation to the other are lacking.

We have therefore, performed a retrospective analysis of patients with gastrointestinal, rheumatic or dermatological immune mediated diseases receiving IFX-IV for maintenance of remission that were switched to IFX-SC based on their physician’s assessment. We report on the efficacy and safety of this elective switching to IFX-SC in patients with CD, UC, SpA, RA, PsA and PsO that were on stable maintenance therapy with IFX-IV.

## Methods

### Study design

This was a multicenter retrospective study in which we analyzed data collected from consecutive patients with immune mediated diseases treated in a hospital setting. Each hospital department was considered a study center and overall, 8 gastroenterology departments, 6 rheumatology departments and 1 dermatology department from 13 Greek hospitals, that are all referral centers, recruited patients for the study.

### Study population

The study population consisted of adult patients (18 years and over) with immune mediated diseases, i.e., Crohn’s disease (CD), ulcerative colitis (UC), spondyloarthritis (SpA), rheumatoid arthritis (RA), psoriatic arthritis (PsA) and chronic plaque psoriasis (PsO) who were transitioned from IFX-IV to IFX-SC, at the discretion of the clinician responsible for the care of the patient, after thorough discussion and in agreement with the patient.

Patients were included in the study if they had quiescent disease, as defined by disease specific composite measures that showed clinical remission, inactive disease or low disease activity. Moreover, they should have been on stable and standard maintenance IFX-IV infusions (5 mg/Kg 8-weekly) for at least 12 months prior to switching. Patients on intensified IFX-IV dosing schedule were excluded.

Patients did not have measurements of IFX trough levels or antibodies prior to switch, since these measurements are not reimbursed by the Greek National Health System.

Data including age, sex, date of disease diagnosis, smoking status, use of corticosteroids and/or immunomodulators at the time of switch, duration of IFX-IV administration prior to switch and past medical history were collected for all patients included in the study.

Patients received the first injection of IFX-SC at a dose of 120 mg 8 weeks after the last IFX-IV administration and every 2 weeks thereafter. The efficacy of IFX-SC was evaluated at least 6 months after switching and was defined as maintenance of clinical remission, inactive disease or low disease activity.

### Definitions of clinical remission, inactive disease or low disease activity

#### Gastrointestinal diseases

In CD, clinical remission was defined as a Crohn’s Disease Activity Index (CDAI) < 150 and in UC as a partial Mayo score < 2.

#### Rheumatic diseases

In SpA patients with predominant axial involvement, inactive disease was defined as Axial Spondyloarthritis Disease Activity Score (ASDAS) < 1.3; in SpA patients with predominant peripheral involvement and in PsA patients, clinical remission and low disease activity was defined as Disease Activity in Psoriatic Arthritis (DAPSA) ≤ 4 and ≤ 14 respectively; and finally, in RA patients, clinical remission and low disease activity was defined as Disease Activity Score – 28 joints – CRP (DAS28-CRP) < 2.6 and ≤ 3.2, respectively.

#### Dermatological diseases

In PsO patients clinical remission was defined as a Psoriasis Area Severity Index (PASI) score of 90–100% and a Physician Global Assessment (PGA) score of 0 or 1.

### Outcomes

The primary endpoint was disease deterioration during the follow up period, according to worsening in disease specific composite measures or a consensus about disease deterioration between investigator and patient leading to a major change in treatment. In particular, disease deterioration according to specific composite measures was defined as a CDAI > 150 in patients with CD; as a partial Mayo score > 2 in patients with UC; a DAS28-CRP > 3.2 in patients with RA; a ASDAS > 2.1 in patients with SpA; a DAPSA > 14 in patients with PsA; and finally, a PASI score < 90 and a PGA score > 1 in patients with PsO.

Secondary endpoints included time to disease deterioration, IFX discontinuation, and the occurrence of adverse events either clinical and/or laboratory.

The study protocol conformed to the principles of the Declaration of Helsinki and was approved by the Institutional Review Boards of the participating hospitals. All patients gave their informed consent to participate in the study. The study was partly funded by a grant from the Athenian Gastroenterology Aid.

### Statistical analysis

Statistical analysis was carried out using the JMP Pro for Windows software program, version 17 (Cary, NC, USA). Quantitative variables were expressed as mean and standard deviation (SD) or median and range, depending on the distribution of the data. The paired t-test and the Wilcoxon signed-rank test were used to compare paired data, as appropriate, with significance defined as *p* value < 0.05. All patients received verbal and written information regarding the switching. The study protocol conformed to the principles of the Declaration of Helsinki and was approved by the Institutional Review Board of the participating hospitals. Informed consent was waived because of the retrospective nature of the study.

## Results

Between April 2023 and April 2024, a total of 344 patients were switched from IFX-IV to IFX-SC in the participating study centers. There were 176 males and 168 females with a mean ± SD age of 45.6 ± 23.3 years. The mean duration of treatment with IFX before switching was a mean ± SD 53.7 ± 36.2 months.

One hundred and thirty-six patients had CD (39.5%), 62 UC (18.0%), 52 SpA (15.1%), 38 PsA (11.1%), 7 RA (2.1%), and 44 had PsO (12.8%). There were also 3 patients (0.9%) that had both SpA and CD, 1 patient (0.3%) that had both SpA and UC and another 1 patient (0.3%) that had both PsA and CD.

Eleven patients (3.2%) were receiving combination treatment with an immunomodulator at the time of switch, while 94 patients (27.3%) had used an immunomodulator in the past. Two hundred and thirty-nine patients (69.5%) had never used an immunomodulator for their disease. Two patients (0.6%) were using oral or iv steroids at the time of switch to IFX-SC. Fifty-six patients (16.3%) had received one, 38 patients (11.0%) two, 15 patients (4.4%) three, 1 patient (0.3%) four, 3 patients (0.9%) five and 4 patients (1.2%) six biologic therapies before starting IFX, while the remaining 227 patients (65.9%) were naïve to biologics when they initiated IFX-IV.

The demographic and clinical characteristics of the patients with Inflammatory Bowel Diseases (IBD), rheumatic diseases and PsO are depicted in Tables 1–3 respectively. The mean ± SD or median (range) values of the CDAI for patients with CD, the partial mayo score for patients with UC, the ASDAS for patients with SpA with predominant axial involvement, the DAPSA for patients with SpA with predominant peripheral involvement and for patients with PsA, the DAS28-CRP for patients with RA and the PASI and PGA for patients with PsO are reported before and after switching (at the end of the follow up period) in Tables 4–6.

**Table 1:** Demographic and clinical characteristics of patients with IBD at the time of switching.

Total number of patients, *n*	203
Crohn’s disease, *n* (%)	140 (68.9)
Ulcerative disease, *n* (%)	63 (31.0)
Age, years mean (SD)	39 (21)
Male/Female, *n* (%)	98 (48.3)/105 (51.7)
Smoking, *n* (%)	59 (29.1)
Disease duration, months mean (SD)	91 (74)
Time of IFX-IV administration, months mean (SD)	52 (23)
Corticosteroid use, *n* (%)	0 (0.0)
Immunomodulator use, *n* (%)	2 (0.9)
Previous biologics, *n* (%)	
One	23 (11.3)
Two	5 (2.4)

**Table 2:** Demographic and clinical characteristics of patients with rheumatic diseases at the time of switching.

Total number of patients, *n*	102
Spondyloarthritis, *n* (%)	56 (54.9)
Psoriatic arthritis, *n* (%)	39 (38.2)
Rheumatoid arthritis, *n* (%)	7 (6.8)
Age, years mean (SD)	44 (19)
Male/Female, *n* (%)	58 (56.8)/44 (43.1)
Smoking, *n* (%)	33 (32.3)
Disease duration, months mean (SD)	104 (81)
Time of IFX-IV administration, months mean (SD)	61 (34)
Corticosteroid use, *n* (%)	2 (1.9)
Immunomodulator use, *n* (%)	9 (8.8)
Previous biologics, *n* (%)	
One	23 (22.5)
Two	11 (10.7)
Three	7 (6.8)
Four	1 (0.9)
Five	3 (2.9)
Six	4 (3.9)

**Table 3:** Demographic and clinical characteristics of the patients with plaque psoriasis at the time of switching.

Total number of patients, *n*	
Plaque psoriasis	44
Age, years mean (SD)	55 (13)
Male/Female, *n* (%)	23 (52.3)/21 (47.7)
Smoking, *n* (%)	13 (29.5)
Disease duration, months mean (SD)	93 (66)
Time of IFX-IV administration, months mean (SD)	37 (18)
Corticosteroid use, *n* (%)	0 (0.0)
Immunomodulator use, *n* (%)	0 (0.0)
Previous biologics, *n* (%)	
One	10 (22.7)
Two	22 (50.0)
Three	8 (18.2)

**Table 4:** Montreal classification and mean ± SD or median (range) values of the CDAI for patients with CD and the partial mayo score for patients with UC, before and after switching (at the end of the follow up period).

Crohn’s disease (Montreal classification)	
Location, *n* (%)	
L1	55 (39.3)
L2	41 (29.3)
L3	44 (30.7)
Behavior, *n* (%)	
B1	128 (91.4)
B2	8 (5.7)
B3	4 (2.8)
Perianal disease, *n* (%)	21 (15.0)
CDAI before/after switching, mean ± SD	101 ± 24/98 ± 28*
Ulcerative colitis (Montreal classification), *n* (%)	
E2	26 (41.2)
E3	37 (58.7)
Partial Mayo score before/after switching, median (range)	0 (0–1)/0 (0–1)*

^*^p was not statistically significant for all variables tested.

**Table 5:** Mean ± SD or median (range) values of the ASDAS for patients with SpA with predominant axial involvement, the DAPSA for patients with SpA with predominant peripheral involvement and for patients with PsA, and the DAS28-CRP for patients with RA before and after switching (at the end of the follow up period).

Spondylarthritis patients with predominant axial involvement	
Inactive disease	36 (35.3)
ASDAS before/after switching, mean±SD	0.79 ± 0.33/0.81 ± 0.29*
Spondylarthritis patients with predominant peripheral involvement	
Clinical remission	17 (16.6)
Low disease activity	3 (2.9)
DAPSA before/after switching, median (range)	3 (1–12)/3 (1–12)*
Psoriatic arthritis	
Clinical remission	31 (30.3)
Low disease activity	8 (7.8)
DAPSA before/after switching, median (range)	3 (1–14)/3 (1–14)*
Rheumatoid arthritis	
Clinical remission	6 (5.9)
Low disease activity	1 (0.9)
DAS28-CRP before/after switching, mean ± SD	1.73 ± 0.85 / 1.69 ± 0.87*

*p was not statistically significant for all variables tested.

**Table 6:** Mean ± SD or median (range) values of the PASI and PGA for patients with PsO before and after switching (at the end of the follow up period).

Inactive disease, *n* (%)	44 (100)
PASI before/after switching, mean ± SD	0.95 ± 0.04/0.96 ± 0.03*
PGA before/after switching, median (range)	0 (0–1)/0 (0–1)*

*p was not statistically significant for all variables tested.

After a mean ± SD follow up period of 8 ± 4 months, 12 patients (3.5%) discontinued treatment with IFX-SC. The mean ± SD time for IFX discontinuation was 11± weeks. Five of these 12 patients (1.5%) discontinued treatment because of disease deterioration and the remaining 7 patients (2.0%) discontinued treatment because of the occurrence of side effects. Of note 3 out of the 5 patients that discontinued treatment because of disease deterioration had both CD (2 patients) or UC (1 patient) and SpA. The clinical characteristics and side effects of the above-mentioned 12 patients are summarized in Table 7.

**Table 7:** Clinical characteristics of the patients that discontinued treatment with IFX-SC.

	Disease	Previous biologics	Reason for discontinuation	Action taken
1	SpA	ADA	Diarrhea	Switch to an agent with different mode of action
2	RA	ADA UPA ETN	Liver function tests	Switch to an agent with different mode of action
3	SpA	CZP	Disease worsening	Switch to an agent with different mode of action
4	CD	Naive	Bruising to lower limbs	Switch to IFX-IV
5	CD	Naive	Allergic reaction	Switch to an agent with different mode of action
6	CD	Naive	Psoriasiform dermatitis	Switch to an agent with different mode of action
7	CD + SpA	Naive	Disease worsening	Switch to an agent with different mode of action
8	CD + SpA	Naive	Disease worsening	Switch to an agent with different mode of action
9	UC + SpA	Naive	Disease worsening	Switch to an agent with different mode of action
10	PsA	CZP	Disease worsening	Switch to an agent with different mode of action
11	UC	Naive	Allergic reaction	Switch to an agent with different mode of action
12	CD	Naive	Lung infection	Switch to an agent with different mode of action

ADA, adalimumab; UPA, upadacitinib; ETN, etanercept; CZP, certolizumab pegol.

The remaining 332 patients (96.5%) showed excellent tolerance of the scheduled treatment, no unscheduled visits were requested, no clinical or laboratory adverse events occurred, and no escalation of treatment was needed.

## Discussion

In this multi-center study, 344 patients with quiescent Crohn’s disease, ulcerative colitis, spondyloarthritis, rheumatoid arthritis, psoriatic arthritis and chronic plaque psoriasis were switched from scheduled maintenance therapy with IFX-IV to IFX-SC. After a mean ± SD follow up period of 8 ± 4 months, the great majority of patients (96.5%) maintained clinical remission, inactive disease or low disease activity and did not develop clinical or laboratory adverse events.

IFX was the first biological therapy employed for the treatment of UC and CD and still has a leading therapeutic position in treatment algorithms for moderate-to-severe disease, refractory to conventional treatment with steroids and/or immunosuppressives, according to the European Crohn’s and Colitis Organization (ECCO) guidelines (7, 8). The same holds true for patients with SpA, PsA and RA according to the European Alliance of Associations for Rheumatology (EULAR) recommendations (9–11), while it is a preferred option for the treatment of PsO (12).

IFX therapy is associated with a considerable financial burden for health-care systems globally (13). Scheduled IFX-IV treatment is associated with higher rates of absenteeism, work-load burden to infusion units, higher rates of infusion-related allergic reactions, loss of response, and need for treatment escalation (14). Furthermore, patients on IFX-IV maintenance therapy are also exposed to the risks of a hospital environment, such as high microbial resistant strains or infectious diseases, something that proved particularly worrisome during the COVID-19 pandemic (15). This study provides evidence that switching patients with quiescent immune mediated disease from IFX-IV to IFX-SC is a safe and effective alternative and to our knowledge this is the first study to address this in such a broad and diverse spectrum of diseases in clinical remission. For health-care systems and infusion units, this strategy lightens up the hospital personnel workload, reduces considerably the all-source cost of treatment and gives independence to the patients (16).

Real world data related to the transition of patients with IBD have already demonstrated that these patients can be effectively switched from IFX-IV to 120 mg fortnightly SC injections even if they are on a dose-escalated IFX administration or with active disease at the time of switch. According to the results of a recent publication, 27 patients with CD and 13 with UC were switched from IFX-IV to IFX-SC and the overall relapse rate was 11% after a 6-month follow up period, even though 42% of the patients were receiving intensified IFX-IV dose (17). In a more recent multicenter observational study in France that included 128 IBD patients who were in steroid free clinical remission and were switched from IFX-IV to IFX-SC, rates of relapse were 13.8, 18.4, 35.3 and 86.7% in those receiving 5 mg/kg/8 weeks, 10 mg/kg/8 weeks, 10 mg/kg/6 weeks and 10 mg/kg/4 weeks at baseline, respectively, after a median follow-up period of 18 months (18). Another multicenter cohort study evaluated 181 patients who switched from IFX-IV to IFX-SC over 1 year. The majority were on 8-weekly dosing of IFX-IV prior to switching and in clinical remission (19). The recently published results of the international multi-center TIME study, in which 231 IBD patients successfully switched from IFX-IV to IFX-SC and from vedolizumab IV to its SC formulation, further strengthen the findings of the studies reported so far (20). Similar results are reported in two recent publications, in patients with CD or UC receiving IFX-IV loading doses who were then randomized to switch to IFX-SC or continue IFX-IV (21) and in patients with active UC were switched to IFX-SC because of IFX-IV secondary loss of response (22).

Less data exists for patients with RA and PsA, while data for patients with SpA or PsO is lacking. In a recent publication, 343 patients with active RA and inadequate response to methotrexate received IFX-IV as induction and were then randomized (1:1) to IFX-SC 120 mg every 2 weeks or IFX-IV 3 mg/kg every 8 weeks thereafter (maintenance). Patients randomized to IFX-IV switched to IFX-SC from week 30 to 54. IFX-SC showed improved efficacy at week 30 compared with IFX-IV, and the between-group difference in efficacy outcomes at week 54 after switching supported the results suggesting an added benefit of IFX-SC compared to IFX-IV at week 30 (23). In a case series of patients with PsA consistently positive clinical outcomes were observed after switching from IFX-IV to IFX-SC, suggesting that patients receiving IFX-IV should be offered a switch to IFX-SC (24).

Among the strengths of our study is its multicenter design and the inclusion of many patients with different immune mediated diseases receiving IFX for maintenance of remission. The findings of our study further confirm and expand previous observations including patients suffering with a broad range of immune mediated diseases in a real-world everyday clinical setting. Most of the patients included had a diagnosis of IBD, because more Gastroenterology centers participated in the study, but also because IFX is one of the drugs still used as first line advanced treatment in these patients, despite the advent of new biologic therapies. Therefore, more IBD patients are receiving IFX compared to patients with SpA, RA, PsA or PsO, for whom many more different biologic agents are available and have superseded IFX as a first line option. According to our data the rate of IFX discontinuation after the elective switch from the IV to the SC formulation was only 3.8% primarily because all our patients had quiescent disease at the time of switching. Our results do demonstrate that patients can be kept away from conceivable unsafe hospital environments, in the era of the available IFX-SC formulation medical prescription.

Limitations of our study, apart from its retrospective nature, could be the small number of patients with skin diseases and the lack of a control group. It could also be argued that patients were not tested for IFX serum concentration or the presence of antibodies before switching; Nevertheless, this study mirrors clinical practice in several countries where these tests are not reimbursed or are not readily available in all hospital settings. Finally, concerning the patients with quiescent CD and UC (i) no histological outcomes were included in the present analysis and (ii) it wasn’t possible to perform fecal calprotectin measurements in all our patients, because this test is also not reimbursed. Acknowledging the aforementioned limitations, our study still provides valuable insights into the efficacy as well as safety of the IFX-IV switch to IFX-SC under specific conditions, opening up avenues for further investigation. Undoubtedly, larger randomized studies are warranted.

In conclusion, our study indicates that elective switching from IFX-IV to IFX-SC seems to be an effective and safe strategy in real-world every day clinical practice to maintain long-term clinical remission, inactive disease or low disease activity in patients with a wide spectrum of immune-mediated diseases.

## Data Availability

The data analyzed in this study is subject to the following licenses/restrictions: the datasets are available at the participating hospitals. Requests to access these datasets should be directed to nikos.viazis@gmail.com.
